# Molecular Docking and Dynamic Simulation of AZD3293 and Solanezumab Effects Against BACE1 to Treat Alzheimer's Disease

**DOI:** 10.3389/fncom.2018.00034

**Published:** 2018-06-01

**Authors:** Mubashir Hassan, Saba Shahzadi, Sung Y. Seo, Hany Alashwal, Nazar Zaki, Ahmed A. Moustafa

**Affiliations:** ^1^College of Natural Science, Department of Biological Sciences, Kongju National University, Gongju, South Korea; ^2^Institute of Molecular Science and Bioinformatics, Lahore, Pakistan; ^3^Department of Bioinformatics, Virtual University Davis Road, Lahore, Pakistan; ^4^College of Information Technology, United Arab Emirates University, Al-Ain, United Arab Emirates; ^5^School of Social Sciences and Psychology, MARCS Institute for Brain and Behaviour, Western Sydney University, Sydney, NSW, Australia

**Keywords:** Alzheimer's disease, computational modeling, dynamic simulation, AZD3293, solanezumab

## Abstract

The design of novel inhibitors to target BACE1 with reduced cytotoxicity effects is a promising approach to treat Alzheimer's disease (AD). Multiple clinical drugs and antibodies such as AZD3293 and Solanezumab are being tested to investigate their therapeutical potential against AD. The current study explores the binding pattern of AZD3293 and Solanezumab against their target proteins such as β-secretase (BACE1) and mid-region amyloid-beta (Aβ) (PDBIDs: 2ZHV & 4XXD), respectively using molecular docking and dynamic simulation (MD) approaches. The molecular docking results show that AZD3293 binds within the active region of BACE1 by forming hydrogen bonds against Asp32 and Lys107 with distances 2.95 and 2.68 Å, respectively. However, the heavy chain of Solanezumab interacts with Lys16 and Asp23 of amyloid beta having bond length 2.82, 2.78, and 3.00 Å, respectively. The dynamic cross correlations and normal mode analyses show that BACE1 depicted good residual correlated motions and fluctuations, as compared to Solanezumab. Using MD, the Root Mean Square Deviation and Fluctuation (RMSD/F) graphs show that AZD3293 residual fluctuations and RMSD value (0.2 nm) was much better compared to Solanezumab (0.7 nm). Moreover, the radius of gyration (Rg) results also depicts the significance of AZD3293 docked complex compared to Solanezumab through residual compactness. Our comparative results show that AZD3293 is a better therapeutic agent for treating AD than Solanezumab.

## Introduction

The β-site APP cleaving enzyme 1 (BACE1) is among the most significant targets for novel drugs to treat Alzheimer's disease (AD) (Huang et al., 2014). The thinning of lipid bilayer has been observed due to protein amyloid-Aβ (Aβ) accumulation and oxidative stress in AD. One study showed that pathologically thin bilayers may play in Aβ aggregation on neuronal bilayers and pathological lipid oxidation may contribute to Aβ misfolding (Korshavn et al., 2017). The aggregation of Aβ peptides results in cellular toxicities due to the formation of polymorphic oligomers, protofibrils, and fibrils (Kotler et al., 2014; Rajasekhar et al., 2016).

Metals accumulation has also deep effect on brain activity and causes neurodegenerations. For example, copper and zinc are associated with the prevalence of AD. In the brains of AD patients, the copper homeostasis is changed with elevated extracellular and low intracellular copper levels. Animals and cell culture studies reported that increasing intracellular copper can cause AD like symptoms through the accumulation of amyloid plaques and tau phosphorylation. Therefore, by changing the copper homeostasis may results in improved cognitive function in animal models of AD (Filiz et al., 2008).

The first generation of BACE1 inhibitors are peptide-based transition state analogs synthesized on the basis of residual sequence of amyloid precursor protein (APP) cleaved by β-secretase (Sinha et al., 1999; Hong et al., 2002; Vassar, 2014). Multiple drugs and antibodies are under different phases of clinical trials to treat AD such as Astra Zenica BACE1 inhibitor (AZD3293) and Solanezumab. AZD3293 is a potent inhibitor against AD and mostly administered orally. AZD3293 prevents the accumulation of β-amyloid and helps slow or cure AD symptoms (Cebers et al., 2017). Clinical studies showed that in both Phase I and II AZD3293 showed good therapeutical potential against AD. Some reports showed that first-generation BACE1 inhibitors were hampered by blood brain barrier (BBB) penetration (Butini et al., 2013; Oehlrich et al., 2014), whereas AZD3293 remarkably has a good penetration against BBB in humans (Cebers et al., 2017).

AZD3293 targets BACE1 and helps reduce Aβ peptide generation (Vassar, 2014). One study showed that AZD3293 has direct effects on plasma and CSF Aβ levels in healthy young and elderly individuals having age 18–55 and 55–80 years, respectively (Alexander et al., 2014). Phase 1 results showed that AZD3293 is well tolerated with no serious adverse effects observed up to the 750 mg in single ascending dose (SAD) study (Alexander et al., 2014). Moreover, a multiple ascending dose (MAD) study reports showed that AZD3293 reduces the CSF Aβ40 and Aβ42 concentrations up to 50 to 75% at 15 or 50 mg doses (Hoglund et al., 2014). Presently, Phase 1 studies of AZD3293 in healthy subjects and in AD patients have been completed, and combined Phase 2/3 trials in 1,551 mild cognitive impairment (MCI) and mild AD patients are planned at different doses from 20 to 50 mg, for 104 weeks duration. Recently, Astra Zenica and Lilly entered into a partnership to jointly develop AZD3293 to treat AD.

Solanezumab (Eli Lilly) is the leading clinical antibody targeting amyloid peptides. Presently, it has been under Phase III clinical trials for the prevention of AD. Solanezumab, a monoclonal antibody IgG1 binds with amyloid-β peptides that aggregate and form plaques in the brain which are considered as basic pathological feature of AD (Villemagne et al., 2013). Solanezumab binds specifically at the monomeric amyloid-β, motif KLVFFAD (Crespi et al., 2015) with pico-molar affinity (Watt et al., 2014). The biding epitope of amyloid-β is known as the nucleation site for Aβ oligomerization, and it is these oligomers of Aβ that are thought to be toxic to neurons. Solanezumab is thought to act as an amyloid beta sink that is “facilitating flux of amyloid beta from a central to peripheral compartment” (DeMattos et al., 2001). Amyloid β-plaques mostly consist of amyloid β-42. Solanezumab binds to free amyloid beta which causes amyloid β-42 to solubilize to re-establish the equilibrium in the cerebrospinal fluid (Farlow et al., 2012). Phase 1–3 results of Solanezumab showed good results against mild to moderate AD.

In the present study, we use docking and dynamic simulation approaches to study the potential of both drugs to cure AD. Research data showed the significance of our proposed methodology in the prediction of drug designing by target various enzymes (Hassan et al., 2017, 2018a,b). We selected 79 drugs and antibodies collectively from clinical drug database (https://clinicaltrials.gov/) and classified them on the basis of active clinical phases. AZD3293 and Solanezumab were selected and further analyzed using various computational tools to calculate their pharmacokinetic properties. Structure-based analyses were performed to evaluate the Root Mean Square Deviation and Fluctuations (RMSD/F), Radius of gyration (Rg) and Solvent Accessible Surface Area (SASA) through dynamic simulation.

## Methodology

### Repossession of target proteins structure

The human crystal structure of beta-secretase (BACE1) and mid-region amyloid-beta (Aβ) in complex with Solanezumab (PDBIDs: 2ZHV & 4XXD), respectively were retrieved form the Protein Data Bank (PDB) (http://www.rcsb.org). The energy minimization of target proteins was conducted by using online tool Chiron to resolve the steric clashes from protein structures (Ramachandran et al., 2011). The stereo-chemical properties, Ramachandran graph and values (Lovell et al., 2003) of targeted proteins were assessed by MolProbity server (Chen et al., 2010), whereas the hydrophobicity graphs were generated by Discovery Studio 4.1 Client (Studio Discovery, 2008). The protein architecture and statistical percentage values of helices, β-sheets, coils and turns were accessed by using the online server VADAR 1.8 (Willard et al., 2003).

### Candidate structure

The AZD3293 and Solanezumab were selected as clinical drugs for present study. The AZD3293 drug was sketched in drawing ACD/ChemSketch tool. 79 drugs and antibodies were selected from clinical drug database (https://clinicaltrials.gov/) and classified on the basis of active clinical phases (Table S1). The selected drug molecule further minimized by UCSF Chimera 1.10.1 (Pettersen et al., 2004). Multiple online drug assessment computational tools such as Molinspiration (http://www.molinspiration.com/) and Molsoft (http://www.molsoft.com/) were used to predict the drug-likeness and biological properties of AZD3293 molecule. Lipinski's rule of five was analyzed using Molsoft and Molinspiraion tools. Furthermore, their predicted Absorption, Distribution, Metabolism, Excretion and Toxicity (ADMET) properties were evaluated by pkCSM online tool (Pires et al., 2015). Solanezumab is a monoclonal IgG1 antibody which is directed against Aβ peptide. The three dimensional (3D) structure of Solanezumab was also accessed from protein data bank as mentioned above.

### Molecular docking simulation

Molecular docking of AZD3293 drug against BACE1 was carried out using diverse AutoDock 4.2 tool according to the specified instructions (Morris et al., 2009). In brief, for receptor protein, the polar hydrogen atoms and Kollman charges were assigned. For ligand, Gasteiger partial charges were designated and non-polar hydrogen atoms were merged. All the torsion angles for AZD3293 were set free to rotate through docking experiment. A grid map of 80 × 80 × 80 Å was adjusted on whole protein structure to generate the grid map and to get the best conformational state of docking. The 100 number of runs were adjusted using docking experiments. The Lamarckian genetic algorithm (LGA) and empirical free energy function were applied by taking docking parameters default. All the docked complexes were further evaluated on lowest binding energy (Kcal/mol) values and hydrogen and hydrophobic interactions analysis using Discovery Studio (2.1.0) and UCSF Chimera 1.10.1. The two dimensional graphical depiction of best docked complexes were accessed by LIGPLOT tool (Wallace et al., 1995).

### Molecular dynamics simulations

Based on docking results, we performed structural dynamic analysis studies on the selected lowest energy valued and best posed docking complex. The MD simulations were carried out by Groningen Machine for Chemicals Simulations (GROMACS) 4.5.4 package (Pronk et al., 2013) with GROMOS 96 force field (Chiu et al., 2009). The PRODRG Server was employed to generate ligands topology files (Schüttelkopf and van Aalten, 2004). Before minimization, the overall system charge was neutralized by adding ions. The energy minimization (nsteps = 50,000) was conducted using the steepest descent approach (1,000 ps) for each protein-ligand complex. The Particle Mesh Ewald (PME) method was employed for energy calculation and for electrostatic and Van der Waals interactions; cut-off distance for the short-range VdW (rvdw) was set to 14 Å, where Coulomb cut-off (r coulomb) and neighbor list (rlist) were fixed at 9 Å (Wang et al., 2010). It permits the use of the Ewald summation at a computational cost comparable with that of a simple truncation method of 10 Å or less, and the linear constraint solver (LINCS) (Amiri et al., 2007) algorithm was used for covalent bond constraints and the time step was set to 0.002 ps. Finally, 20 ns molecular dynamics simulation was carried out for all the complexes with nsteps 1,000,000. The Root Mean Square Deviation and fluctuation (RMSD/F), Soluble Accessible Surface Area (SASA) and Radius of gyration (Rg) analysis were carried out using Xmgrace (http://plasma-gate.weizmann.ac.il/Grace/) and UCSF Chimera 1.10.1 software.

## Results and discussion

### Structural assessment of BACE1, Aβ, and solanezumab

BACE1 is a class of hydrolase protein comprises having single chain and 411 amino acids. The structural architecture of BACE1 showed that it consists of 9% helices, 49% β sheets, 41% coil, and 21% turns. The X-Ray diffraction study confirmed its resolution 1.85 Å, *R*-value 0.290 and unit cell crystal dimensions like length and angles of coordinates for a = 102.33, b = 102.33, and c = 170.52 with angles 90, 90, and 120° and for all α, β, and γ dimensions respectively. The Ramachandran plots and values indicated that 98% of residues were in favored regions and >99.8% residues were present in allowed regions. Ramachandran graph and values showed the good accuracy of phi (ϕ) and psi (ψ) angles among the coordinates of receptor molecules and most of residues plummeted in acceptable region. The Aβ is clump of multiple chains (A, B, C, D, and E) which contain 42 amino acids.

The 3D crystal structure of Solanezumab showed that it consists of two Fab light chains (A, D) and two Fab heavy chains (B, E) having 219 and 223 amino acids, respectively. The X-Ray diffraction study indicated its resolution 2.41Å and R-value 0.290. The unit cell crystal dimensions such as length and angles of coordinates for a = 38.80, b = 73.56, and c = 92.12 with angles α = 109.91°, β = 93.64°, and γ = 93.31° for all dimensions respectively. Moreover, the VADAR 1.8 analysis showed that Solanezumab contain 1% helices, 59% β sheets, 38% coil, and 4% turns. Furthermore, Ramachandran analysis depicts the 98% residues are present in favored region while 9.8% amino acids are lie in allowed region. The graphical depiction of Ramachandran plots of BACE1 and Solanezumab are mentioned in detail in Supplementary Data (Figures S2, S3), respectively. The detailed structural analysis of BACE1, Aβ and Solanezumab are discussed in Table S4.

### Computational evaluation of AZD3293

#### Chemoinformatics properties and Lipinski's rule (RO5) validation of AZD3293

**Figure 1:**
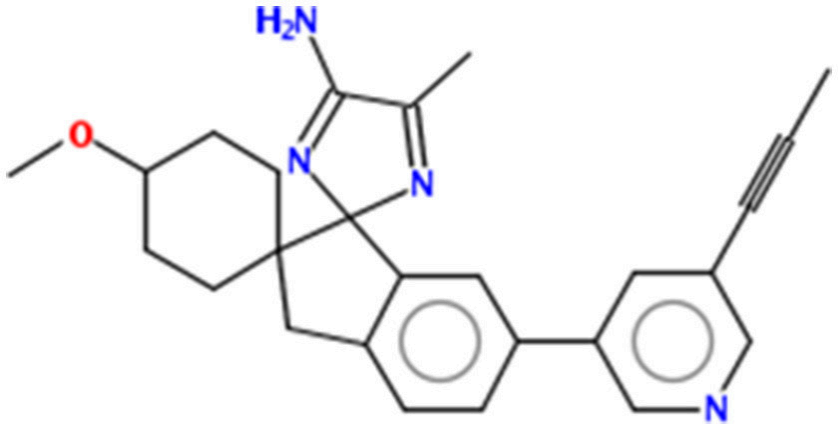
Chemical structure of AZD3293.

Multiple computational approaches were employed to predict basic chemoinformatics and basic molecular properties of AZD3293 (Figure 1). The predicted properties such as molecular weight (g/mol) polar surface area (PSA, A^2^), molar volume (cm^3^), density (cm^3^), molar refractivity (cm^3^) and RO5 were evaluated to justify their drug likeness behavior (Prerana et al., 2015). One study revealed that PSA is a significant parameter for drug absorption prediction in drug discovery (Ertl et al., 2000). The molar refractivity and molecular lipophilicity properties of drug molecules are also important for receptor binding, bioavailability and cellular uptake within the body. One report justifies the standard values for molar refractivity (40–130 cm3) and molecular weight (160 to 480 g/mol) and PSA (<89 Å2) (Ghose et al., 2012). Moreover, the qualifying range for total number of atoms in the drug molecule is between 20 and 70 atoms. Table 1 results showed that molar refractivity and PSA predicted values were comparable with standard values. AZD3293 showed higher molar refractivity and log*P* value (122.60 cm^3^ and 4.82) respectively, compared to standard values. Comparative results showed that AZD3293 confirm its significant and good candidate molecule.

**Table 1:** Chemoinformatic properties of AZD3293.

**AZD3293**	**Properties**
Molecular Formula	C_26_H_28_N_4_O
Molecular Weight (g/mol)	412.52672
Hydrogen Bond Acceptor	4
Hydrogen Bond Donor	2
Rotatable Bonds	2
Log*P*	4.82
No of atoms	31
Polar Surface Area (A^2^)	54.70
Molar Refractivity (cm^3^)	122.60
Density (cm^3^)	1.23
Molar Volume (cm^3^)	333.7
Drug likeness	0.40
Lipinski validation	Yes
GPCR ligand	0.51
Ion channel modulator	0.49
Kinase inhibitor	0.30
Nuclear receptor ligand	0.46
Protease inhibitor	0.53
Enzyme inhibitor	0.56

Furthermore, AZD3293 was validated by RO5 and contains no more and < 10 hydrogen bond acceptors (HBA) and 5 (hydrogen bond acceptors) HBD, respectively. Moreover, the log*P* and molecular mass value also be <5 and 500 (g/mol), respectively. Literature study revealed that the exceed values of HBA and HBD results in poor permeation (Kadam and Roy, 2007). The hydrogen bonding ability has been considered a significant parameter for drug permeability. Our results justified that the AZD3293 possess < 10 HBA, < 5 HBD, < 500 (g/mol) molecular weight and <5 log*P* values which were comparable with standard values. The reported study showed that molecules with poor absorption are more likely to be observed upon Lipinski violation. However, multiple examples are available for RO5 violation amongst the existing drugs (Bakht et al., 2010; Tian et al., 2015). The predicted drug score (0.40) and bioactivity score values are also significant for further analysis. The predicted score values of G-protein couple receptor (GPCR) (0.51), protease and enzymes inhibition score (0.53 and 0.56), respectively showed their good lead like behavior.

#### Pharmacokinetic properties of AZD3293

The designing of novel drugs require a high attention rate with good pharmacokinetic properties. The Absorption, Distribution, Metabolism, Excretion, and Toxicity (ADMET) properties were assessed to confirm the efficacy of candidate molecules. In ADMET evaluation, absorption like water and intestinal solubility (log mol/L & % absorbed) and skin permeability (logKp) predicted values justified the strong therapeutic potential of chemical compounds. One report justified that compounds with good absorption values have potency to cross gut barrier by passive penetration to reach the target molecule (Selick et al., 2002). The water solubility results justified that AZD3293 showed good absorption value (−4.956 log mol/L). Moreover, the intestinal solubility prediction value (96.90) also justified its good efficacy compared to a standard value (>30% abs). Any chemical lead like structure with <30% absorbance value is considered as poorly absorbed compound (Pires et al., 2015). The predicted skin permeability value (−2.902 log Kp) of AZD3293 was also comparable with standard value (−2.5 logKp) which showed their significance as a good lead structures and justified their drug likeness behavior. The p-glycoprotein inhibition behavior was also confirmed for AZD3293. Moreover, in distribution properties, the Blood Brain Barrier (BBB) and Central Nervous System (CNS) permeability values of AZD3293 were also evaluated and compared with the standard values (>0.3 to < −1 log BB and >−2 to < −3 logPS) respectively. It has been observed that compounds with a >0.3 log BB value have potential to cross BBB, while with < −1 value are poor distributed to brain. The predicted results showed that AZD3293 have poor BBB value (−0.164 log BB). However, the CNS permeability value (−1.72 log PS) is quite comparable with standard value. Similarly, the compounds have > −2 logPS value are considered to penetrate the CNS, while with < −3 are difficult to move in the CNS.

Moreover, metabolic behavior of AZD3293 was confirmed by CYP3A4, which is isoform of cytochrome P450. The excretion and toxicity predicted values were also justified the drug likeness behavior of AZD3293 on the basis of total clearance (log ml/min/kg), AMES toxicity, maximum tolerated dose (MTD) and LD_50_ values. The AMES toxicity prediction for AZD3293 also confirmed there is non-mutagenic and non-toxic behavior. The hepatotoxicity positive effect showed its lethal behavior while skin sensitivity negative behavior presents their non-toxic and less sensitive effects. Disruption of normal liver function is commonly associated with hepatotoxicity (Table 2).

**Table 2:** Pharmacokinetic assessment of AZD3293.

**Property**	**Model name**	**Predicted value**
Absorption	Water solubility	−4.956 (log mol/L)
	Intestinal absorption (human)	96.901 (% Absorbed)
	Skin Permeability	−2.902 (log Kp)
	P-glycoprotein substrate	Yes
	P-glycoprotein I inhibitor	Yes
	P-glycoprotein II inhibitor	Yes
Distribution	VDss (human)	0.772 (log L/kg)
	Fraction unbound (human)	0.057 (Fu)
	BBB permeability	−0.164 (log BB)
	CNS permeability	−1.72 (log PS)
Metabolism	CYP2D6 substrate	No
	CYP3A4 substrate	Yes
	CYP1A2 inhibitior	No
	CYP2C19 inhibitior	Yes
	CYP2C9 inhibitior	Yes
	CYP2D6 inhibitior	No
	CYP3A4 inhibitior	Yes
Excretion	Total Clearance	0.469 (log ml)
	Renal OCT2 substrate	Yes
Toxicity	AMES toxicity	No
	Max. tolerated dose (human)	−0.497 (log mg)
	hERG I inhibitor	No
	hERG II inhibitor	Yes
	Oral Rat Acute Toxicity (LD_50_)	2.864 (mol/kg)
	Oral Rat Chronic Toxicity	1.06 (log mg/kg)
	Hepatotoxicity	Yes
	Skin Sensitisation	No
	*T. Pyriformis* toxicity	0.727 (log ug/L)
	Minnow toxicity	0.045 (log mM)

### Molecular docking analysis

#### AZD3293 binding energy analysis against BACE1

The AZD3293-BACE1 docked complexes were analyzed on the basis of lowest binding energy values (Kcal/mol) and hydrogen/hydrophobic interaction analyses. The best pose selection from all the docking complexes was also conducted on the basis of lowest binding energy values and bonding interaction pattern within the active region of target protein. Results showed that AZD3293 with pose 8 was the most active conformational position and predicts the best energy values (−5.33 kcal/mol) as compared to others docking complexes (Table 3). Furthermore, the intermolecular energy value (−7.42 Kcal/mol) was also good compared to other docking pose conformations. The Autodock energy calculation was conducted by using equation 1. However, the standard energy error for Autodock is 2.5 Kcal/mol (Morris et al., 2013). In all docking complexes poses the binding energy difference was less 2.5 Kcal/mol.

**Table 3:** Docking energy of AZD3293 against BACE1.

**Docking Poses**	**Binding energy (Kcal/mol)**	**Intermol. energy (Kcal/mol)**	**Internal energy (Kcal/mol)**	**Torsional energy (Kcal/mol)**	**Unbound energy (Kcal/mol)**
Docking Pose-1	−4.41	−6.50	−0.69	2.09	−0.69
Docking Pose-2	−4.87	−6.96	−0.37	2.09	−0.37
Docking Pose-3	−3.58	−5.67	−0.7	2.09	−0.7
Docking Pose-4	−4.79	−6.88	−0.5	2.09	−0.5
Docking Pose-5	−4.03	−6.12	−0.67	2.09	−0.67
Docking Pose-6	−4.42	−6.51	−0.71	2.09	−0.71
Docking Pose-7	−4.08	−6.17	−0.71	2.09	−0.71
Docking Pose-8	−5.33	−7.42	−0.64	2.09	−0.64
Docking Pose-9	−4.49	−6.58	−0.54	2.09	−0.54
Docking Pose-10	−3.71	−5.80	−0.65	2.09	−0.65

Δ*Gbinding* = Δ*Ggauss* + Δ*Grepulsion* + Δ*Ghbond* + Δ*Ghydrophobic* + Δ*Gtors*…..(i)

Here, ΔG gauss: attractive term for dispersion of two gaussian functions, ΔGrepulsion: square of the distance if closer than a threshold value, ΔGhbond: ramp function-also used for interactions with metal ions, ΔGhydrophobic: ramp function, ΔGtors: proportional to the number of rotatable bonds.

#### Binding conformational analysis

**Figure 2:**
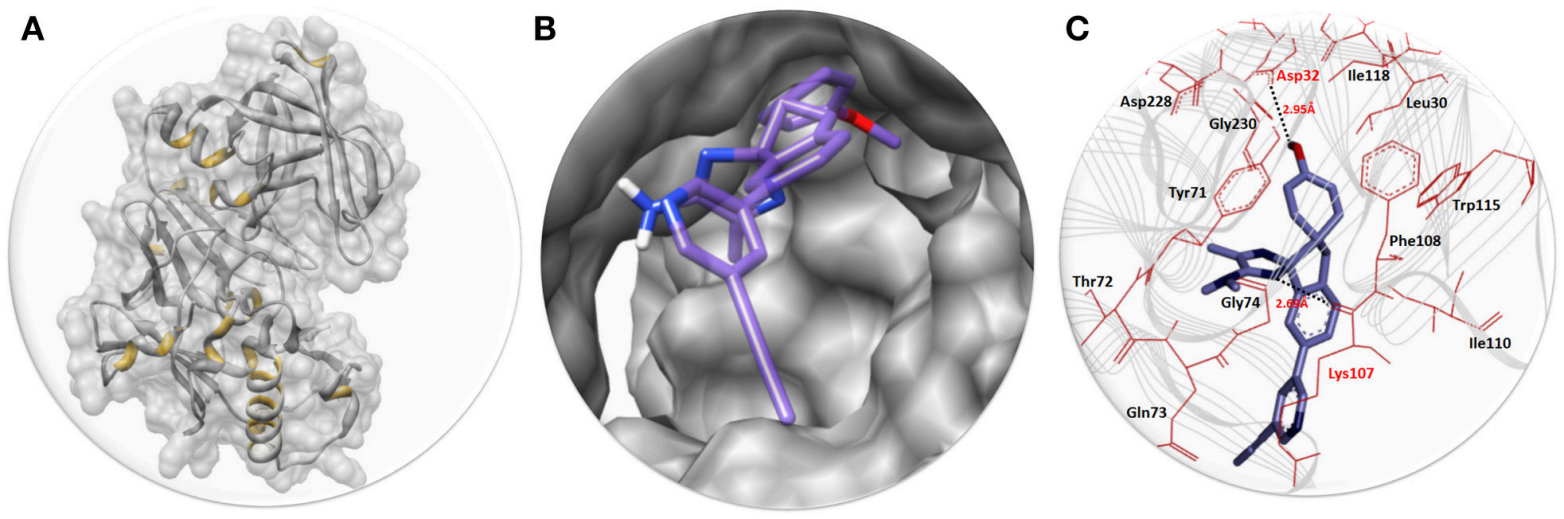
**(A)** The BACE1 3D structure in gray color with interior yellow color having surface format. **(B)** The close depiction of active region of target protein with embedded AZD3293. The AZD3293 is mentioned in purple color while the functional groups such as amino and oxygen are highlighted with blue and red colors, respectively. **(C)** Docking interaction between AZD3293 and target protein (BACE1). The target protein is highlighted in line ribbon format with gray color. The active binding amino acids are highlighted in dark maroon color around the ligand. Two hydrogen bonds were observed between AZD3293 and BACE1 amino acids at Asp32 and Lys107 with distances 2.95 and 2.68 Å, respectively. The black dotted lines show the binding distance in angstrom (Å).

The best docked energy complexes were further deep analyzed on the basis of hydrogen and hydrophobic interactions pattern between ligand and target protein. The active binding region of BACE1 was accessed through one study (Hernández-Rodríguez et al., 2016). Results showed that AZD3293 perfectly binds within the active region of target protein by forming couple of hydrogen bonds. The graphical depiction of best docking complex along with receptor and active binding pocket region is mentioned in Figure 2.

The structure activity relationship (SAR) analysis shows that AZD3293 forms two hydrogen bonds at specific residues (Asp32 and Lys107) with target protein. The hydroxyl group of benzene ring of AZD3293 interacts with Asp32 having bonds length 2.95 Å. Similarly, amino group of other AZD3293 forms another hydrogen bond with bond length 2.68 Å. One study also justified that these interacted residues are significant in the downstream signaling pathways (Hernández-Rodríguez et al., 2016). The graphical representations of all other docking poses are mentioned in Supplementary Data (Figures S5–S13).

#### Binding analysis of solanezumab with Aβ

**Figure 3:**
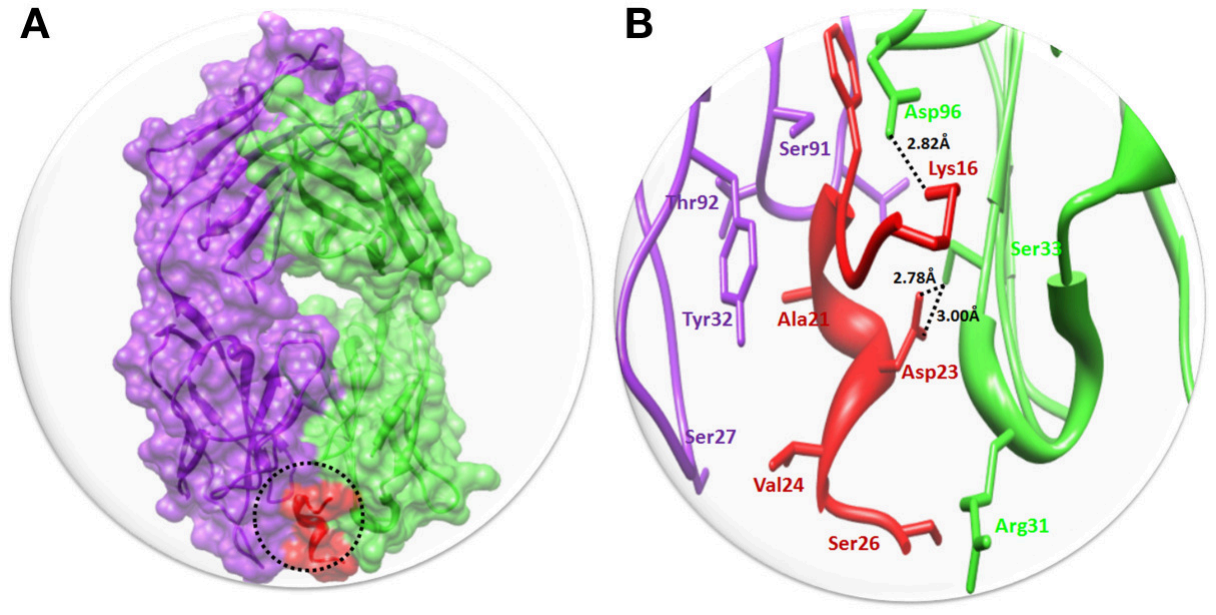
The binding interaction of Solanezumab with Aβ. **(A)** The complex structure of Solanezumab with Aβ. The light chain is represented in purple color while heavy chain is highlighted in green color. Aβ is depicted in red color at Fab region of Solanezumab. The whole structure is represented in surface format. **(B)** The epitope region residues of Solanezumab of both light and heavy chain are represented in purple and green colors, respectively. The Aβ interacted residues are represented in red color while the black dotted lines are for bonding distance in angstrom (Å).

Solanezumab is a monoclonal IgG1 antibody directed against Aβ peptide. Solanezumab exerts its effect by sequestering Aβ, shifting equilibria between different species of Aβ, and removing small soluble species of Aβ that are directly toxic to synaptic function. The binding interaction pattern was observed to residual involvement of both proteins. The results showed that Fab region of both light and heavy chains of Solanezumab collapse with Aβ peptide. However, the observed interacted residue were present at heavy chain. The residues of Aβ peptide such as Lys16 and Asp23 are directly involved in hydrogen bonding with heavy chain of Asp96 and Ser33 respectively. The Lys16 forms hydrogen bond with Asp96 with distance 2.82 Å. While Ser33 forms two hydrogen bonds with Asp23 with bond length 2.78 and 3.00 Å, respectively (Figure 3). The Aβ peptide region is considered as most active part and multiple hydrophobic interaction were also observed against Solanezumab. Literature report also justified that peptide region from Lys16 to Val24 is most significant in the binding with Solanezumab and further downstream signaling pathways (Crespi et al., 2015). The hydrophobic interacted residues of Solanezumab are as follows: Phe27, Phe55, Phe36, Ser94, Gly95, His34, Tyr32, Lys50, Asp28, Thr92, and Val94 which binds with Aβ peptide. The hydrophobic interacted residues of both Solanezumab and Aβ peptide are mentioned in Supplementary Data (Figure S14).

#### Dynamical cross-correlation matrix (DCCM) of BACE1 and solanezumab

**Figure 4:**
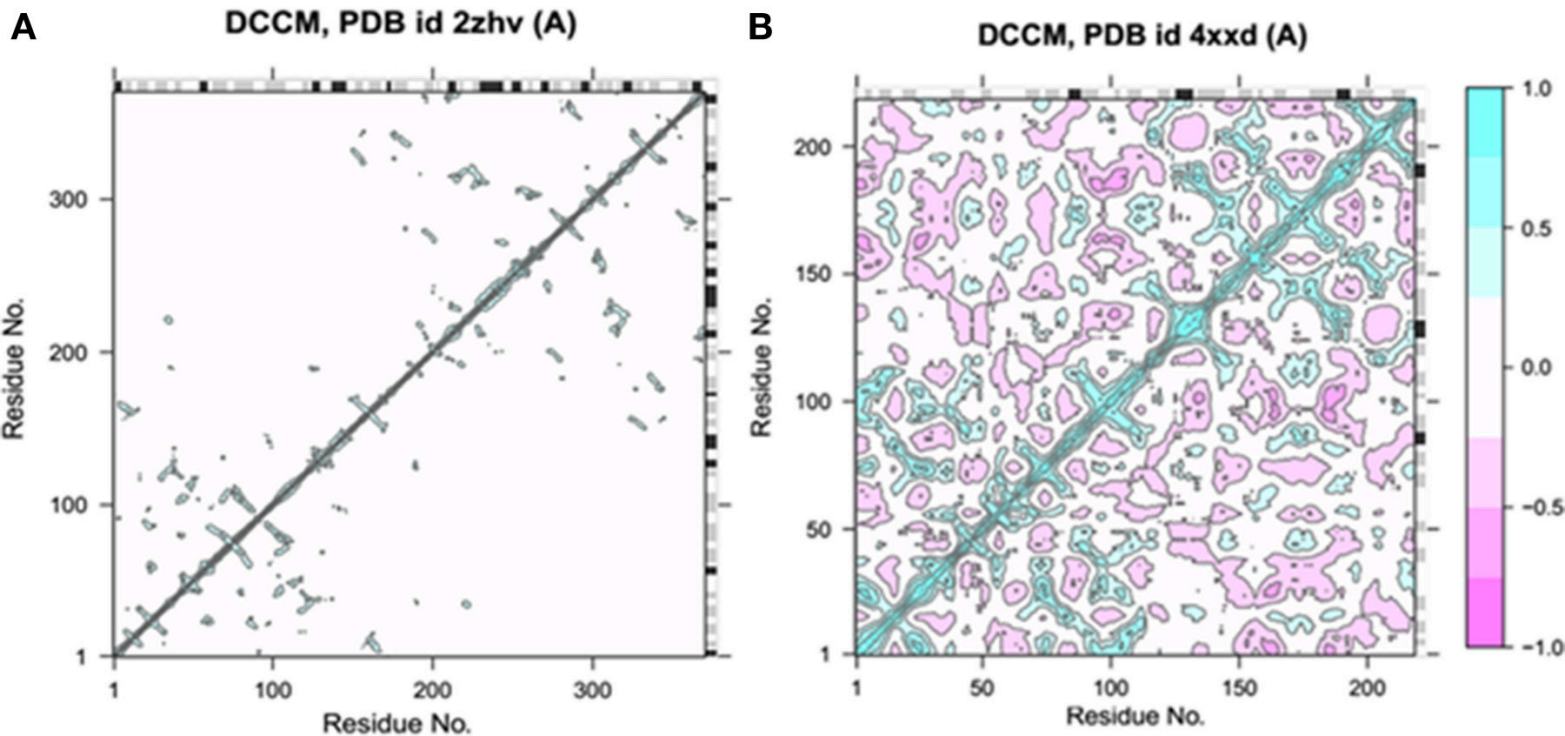
Dynamical cross-correlation analysis of both target structures. **(A)** The residual cross-correlation analysis of BACE1 (PDBID: 2ZHV). **(B)** The residual cross-correlation analysis of solanezumab antibody (PDBID: 4XXD).

Bio3D-web is an online application to analyze the sequence, structure and conformational heterogeneity of protein families (Skjærven et al., 2016). The residual fluctuations of target protein structures were analyzed using Bio3D server. The generated dynamical cross-correlation graphs depicts positive and negative correlation effect of amino acids. The pairwise correlated graphs were generated between the function of residue indices i and j. The predicted map results were analyzed on the basis of colors such as dark cyan, white and pink, respectively. The fully correlated pairs are represented by cyan color, while the anti-correlated are justified as pink color. However, the moderately and un-correlated regions are highlighted by yellow and cyan, respectively. The comparative results showed that BACE1 depicted good residual correlated motions as compared Solanezumab complex (Figure 4).

#### Normal mode analysis (NMA) fluctuations analysis BACE1 and solanezumab

**Figure 5:**
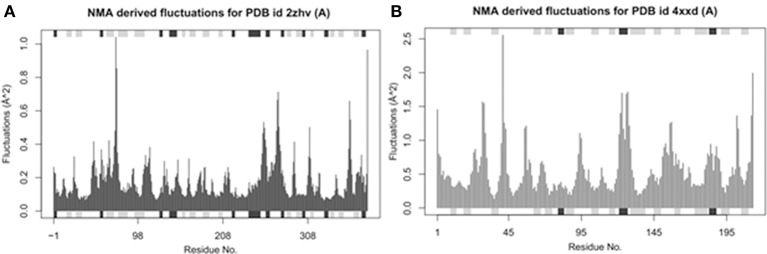
Ensemble normal mode analysis. **(A)** The residual fluctuations analysis of BACE1 (PDBID: 2ZHV). **(B)** The residual fluctuations analysis of solanezumab antibody (PDBID: 4XXD). The fluctuations peaks in both graphs less than <0.05 showed the accuracy of crystal structural behavior.

Bio-3D NMA web analysis displays the dynamic behavior of various residues in the protein structures (Yao et al., 2016). The predicted graphs results showed that BACE1 has less residual fluctuations compared to Solanezumab antibody complex. The comparative analysis reveals that BACE1 residual fluctuation range start from 0 to 1.0, while Solanezumab has 0–2.5. The N-terminus region of BACE1 has less amino acids fluctuations compared to C-terminus. Whereas, in Solanezumab whole structure is fluctuated (Figure 5).

### Molecular dynamic simulations analysis

#### Root mean square deviation and fluctuation of target structures

**Figure 6:**
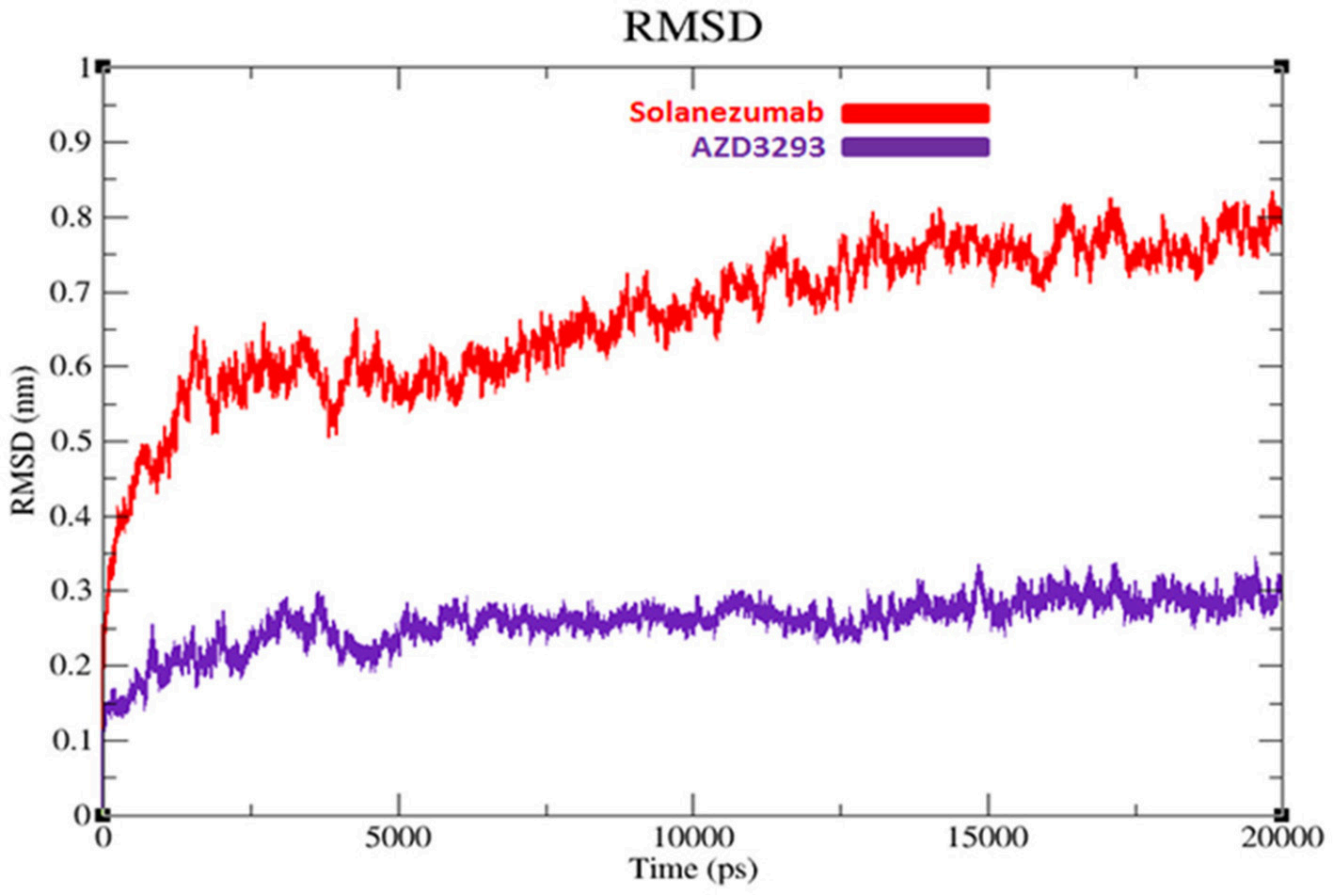
RMSD graphs of AZD3293 and Solanezumab at 20 ns. The graph lines with red and purple represents AZD3293 and Solanezumab complexes, respectively.

**Figure 7:**
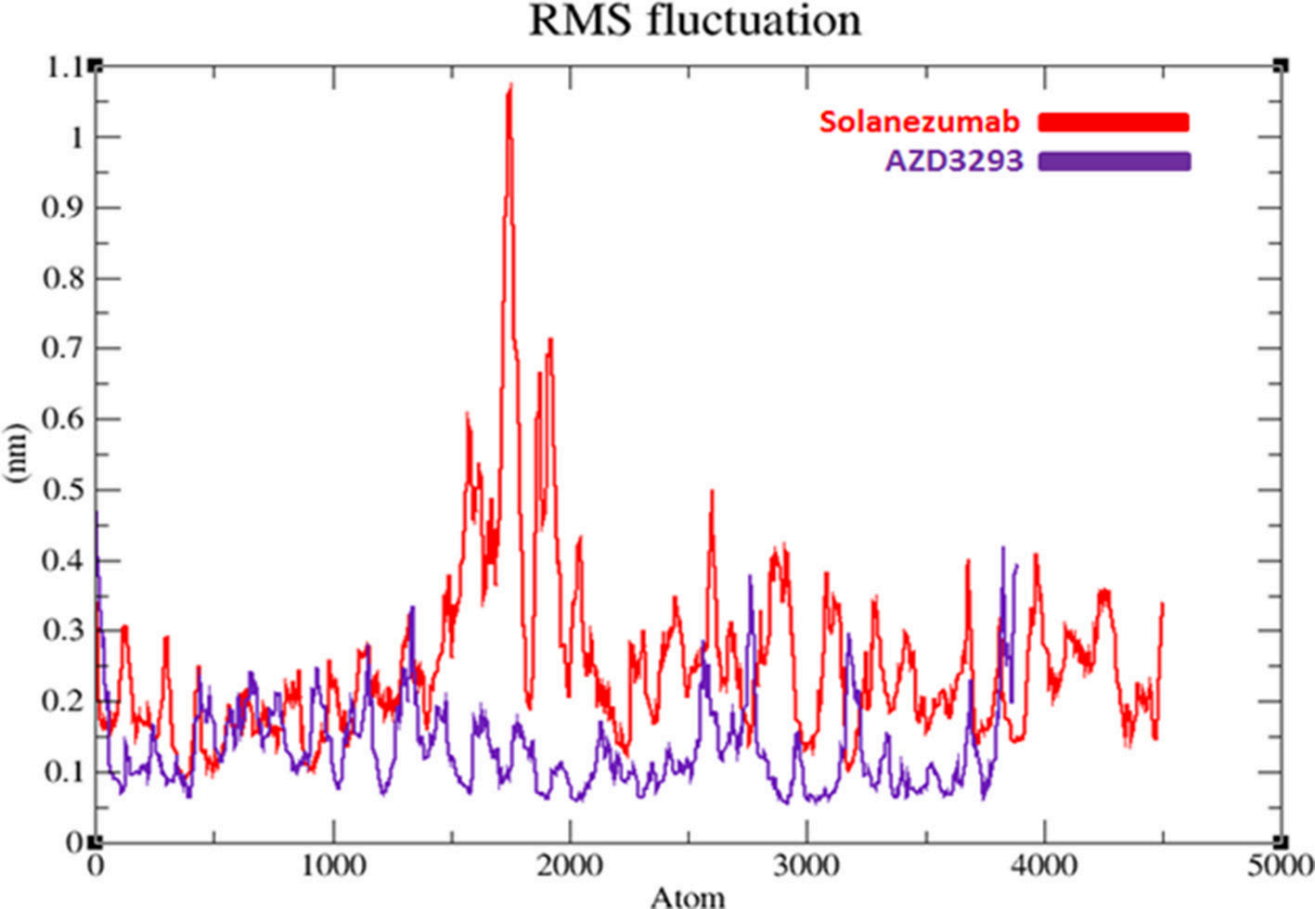
RMSF graphs of AZD3293 and Solanezumab at 20 ns. The graph lines with red and purple represents AZD3293 and Solanezumab complexes, respectively.

To evaluate the flexibility and overall stability of docked complexes, we conducted time dependent MD simulation at 20 ns using Gromacs 4.5.4. The residual deviations and fluctuation in the complexes were determined by using RMSD and RMSF graphs generated by using Xmgrace software. Figures 6, 7 exhibited the residual deviation and fluctuations of AZD3293 and Solanezumab docked complexes, respectively. The increasing trend was observed in both (AZD3293 and Solanezumab) complexes having diverse RMSD values 0–0.25 and 0.5 nm at equilibrium state (starting) from 0 to 2,500 ps in the simulation period. Initially, the AZD3293 complex shows little variations while Solanezumab depicted higher fluctuations. From 2,500 to 5,000 ps, AZD3293 complex indicates little fluctuations while Solanezumab exhibited more deviations with increased value of RMSD value 0.6 nm. From 5,000 to 20,000 ps AZD3293 still remain static depiction with constant RMSD value 0.25 nm while Solanezumab continuously increases with enhanced RMSD value. The overall MD results showed that AZD3293 fluctuations was much better as compared to Solanezumab in the simulation time. The predicted graph suggested that AZD3293 complex is much better and little fluctuated throughout the simulation period. The RMSF results of both AZD3293 and Solanezumab reflect the loops fluctuations throughout the simulation period. The comparative analyses showed that Solanezumab exhibited higher fluctuated peaks as compared to AZD3293 which may depict the significance of AZD3293 over Solanezumab. Insight from MD simulations stable behaviors of AZD3293 docked complex throughout MD trajectories thus increasing the efficacy of docking results.

#### Radius of gyration analyses

**Figure 8:**
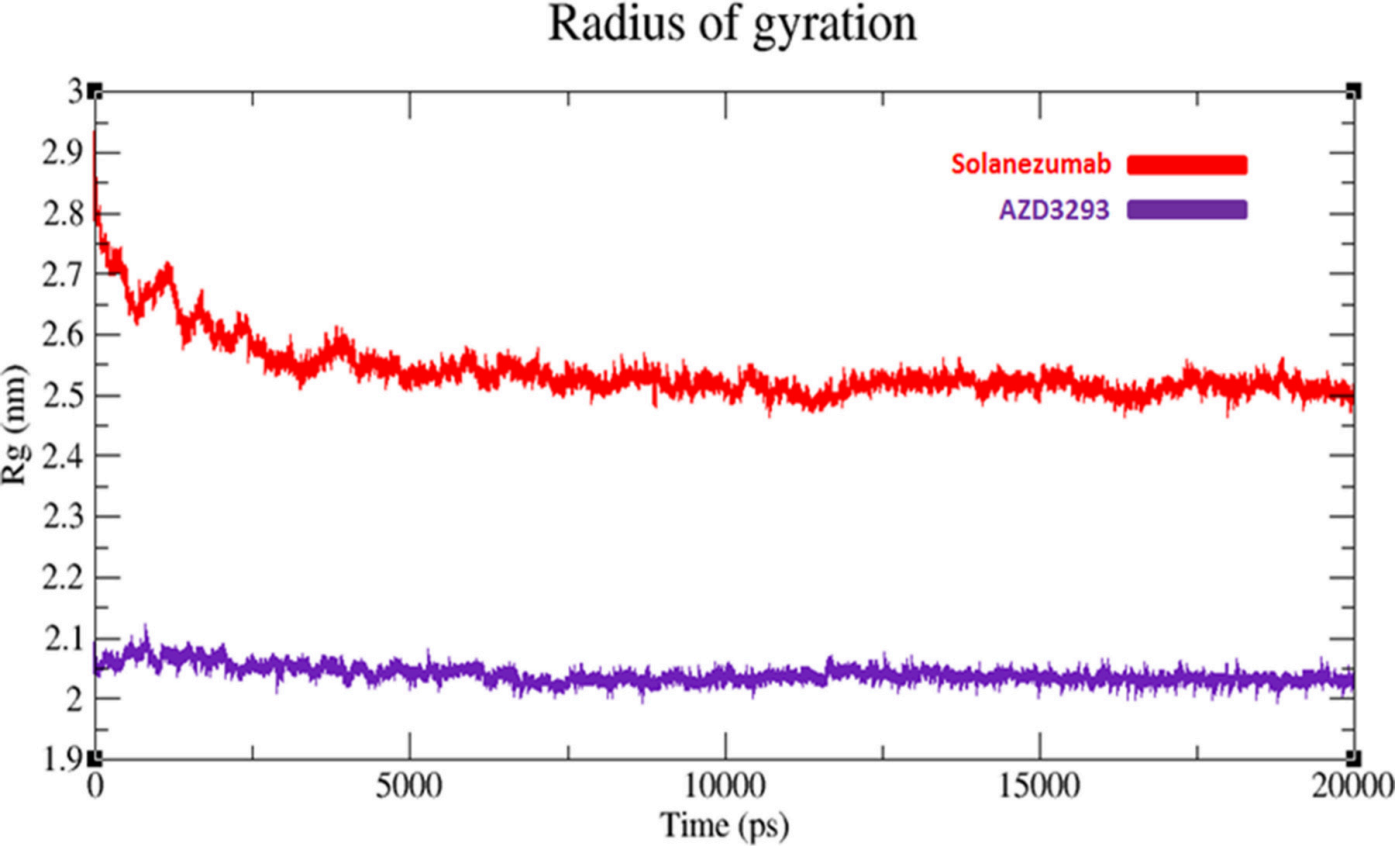
Radius of gyration (Rg) graph of AZD3293 and Solanezumab at 20 ns. The graph lines with red and purple represents AZD3293 and Solanezumab complexes, respectively.

The compactness of protein is measured by radius of gyration (Rg). The predicted results showed that AZD3293 showed much static depiction and constant Rg value at 2.05 nm throughout the simulation time 0–20,000 ps. Whereas, Solanezumab depicts variations at initiating while later shows stability with Rg value 2.5 nm. The comparative results justified that the residual backbone and proper conformation of AZD3293 docked receptor is much batter compared to Solanezumab complex (Figure 8).

The comparative analyses showed that AZD3293 has better therapeutical potential than Solanezumab. Our analyses explore the residual fluctuations after binding the drug with BACE1 receptors. The predicted results depicted the significance of AZD3293 over Solanezumab in treating AD. One limitation with MD simulations is that the protein covalent bonds remain unbroken which may disturb the rmsd and rmsf graph fluctuations. However, there are few things that can done in future work to enhance the accuracy and reliability of MD simulation, such as improvement of simulation time, MD force field, and novel algorithms.

In the future, a molecular dynamics simulation study may be used as good computational approach to study the interactions of Aβ40 and Aβ42 with model neuronal membranes. The higher running time (i.e., 200–300 ns) scale with best force field (i.e., a collection of equations and associated constants designed to reproduce molecular geometry and selected properties of tested structures) simulation can help investigate the effect of Aβ peptides on neuronal membrane. Moreover, some key factors should also be investigated such as surface area per lipid, bilayer thickness and lipid order parameter to obtain the reliable results. The advancement in computer hardware can further mature the simulation method.

## Conclusion

Computational interpretation of newly designed compounds and their binding analysis in the active region of target proteins allow pharmaceutical industries and labs to test the efficacy of drugs before starting experimental lab work. In our *in-silico* approach, we compared two clinical drugs (AZD3293 and Solanezumab) are being testing against AD. Multiple online drug analysis computational tools and server were employed to predict the efficacy and possible lethality of selected compounds. Molsoft and molinspiration results depict the validity of RO5 for AZD3293. Our results show that AZD3293 may be considered as a good therapeutic agent and have good drug like behavior. Moreover, their pharmacokinetic properties such as ADMET properties also justified their good lead like behavior and drug potential. In all parameters such as Absorption, Distribution and Excretion, AZD3293 gave positive results except little lethality in Metabolism and Toxicity. AZD3293 depicts its hepatotoxicity behavior which may cause serious effect in the body. Furthermore, the docking based hydrogen binding and structural analyses show the significance of both selected drugs with proper conformation within the active region of target proteins. The cross correlation and normal mode results depict the structural stability of receptors molecules. The comparative analyses showed that AZD3293-receptor complex has more stable behavior compared to Solanezumab. Finally, a detail simulation study was performed to get the deeper insight of backbone fluctuations and structural stability through RMSD/F results. The generated RMSD/F graphs results showed that the AZD3293-BACE1 docked complex predicts stable behavior compared to Solanezumab regarding the simulation time. The comparative RMSD value (0.2 nm) of AZD3293 was more significant compared to Solanezumab (0.7 nm). The radius of gyration (Rg) results also represent the proper residual conformation and compactness AZD3293 receptor compared to Solanezumab. Based on our computational and comparative results, we may conclude the significance of AZD3293 over Solanezumab on the basis of good binding and structural stability behavior against target proteins of AD.

## Author contributions

MH conceived the study under the guidelines of SYS and AM. MH and SS collected data and performed the experimental work. MH wrote the initial draft of manuscript. HA and NZ make corrections in initial draft. SYS and AM edited the manuscript and compiled it into final format.

### Conflict of interest statement

The authors declare that the research was conducted in the absence of any commercial or financial relationships that could be construed as a potential conflict of interest.
