# Supplementary material for: Molecular Docking and Dynamic Simulation of AZD3293 and Solanezumab Effects Against BACE1 to Treat Alzheimer's Disease
Source: Front Comput Neurosci. 2018 Jun 1;12:34. doi: 10.3389/fncom.2018.00034 (PMC5992503; doi:10.3389/fncom.2018.00034)
Supplement: Supplementary file 1 [file Data_Sheet_1.DOCX]

**Supplementary data**

**Molecular docking and dynamic simulation of AZD3293 and Solanezumab effects against BACE1 to treat Alzheimer’s disease**

**Running Title: Computational analysis of AZD3293 and Solanezumab effect against AD**

**Hassan, M.^1^, Shahzadi S ^2,3^, Seo, SY.^1^, Alashwal, H^4^, Zaki, N^4^., Moustafa, AA^5,6^***

^1^Department of Biology, College of Natural Sciences, Kongju National University, Gongju, 32588, Republic of Korea.

^2^Institute of Molecular Science and Bioinformatics, Lahore, Pakistan & ^3^Department of Bioinformatics, Virtual University Davis Road, Lahore, Pakistan.

^4^College of Information Technology, United Arab Emirates University, Al-Ain, 15551, UAE.

^5^School of Social Sciences and psychology & ^6^MARCS Institute for Brain and Behaviour, Western Sydney University, Sydney, New South Wales, Australia.

**To whom correspondence should be addressed:**

**Ahmed A. Moustafa**,

School of Social Sciences and Psychology & Marcs Institute for Brain and Behaviour, Western Sydney University, Sydney, NSW.

Email: [a.moustafa@westernsydney.edu.au](mailto:a.moustafa@westernsydney.edu.au)

**Keywords**: Alzheimer’s disease, Computational modelling, Dynamic simulation, AZD3293 and Solanezumab

**Table S1.** Drugs that involves in AD extracted from database

| **Sr. no** | **Clinical Drugs** | **Status** | **Phases** |
| --- | --- | --- | --- |
| 1 | AZD3293 | Continued | 2, 3 |
| 2 | Atomoxetine hydrochloride | Completed |  |
| 3 | Huperzine A | Unknown |  |
| 4 | Rofecoxib | Completed |  |
| 5 | Naproxen | Completed |  |
| 6 | Transdermal estradiol | Completed |  |
| 7 | Medroxyprogesterone | Completed |  |
| 10 | Raloxifene | Completed |  |
| 11 | Lecozotan SR | Completed |  |
| 12 | Sertraline | Completed |  |
| 13 | Epigallocatechin-Gallate | Completed |  |
| 16 | Galantamine | Completed |  |
| 17 | NIC5-15 | Completed |  |
| 18 | Donepezil, AZD3480 | Completed |  |
| 19 | JNJ-54861911, 5-25 mg | Continued | 2, 3 |
| 20 | PF-04360365, 7.5-10 mg/kg | Completed |  |
| 21 | Sagramostim | Continued | 2 |
| 22 | Rivastigmine Patch | Continued | 4 |
| 23 | AC-1204 | Continued | 2, 3 |
| 24 | CAD106 Immunotherapy, CNP520 | Continued | 2 |
| 25 | ST101 | Completed |  |
| 27 | Verubecestat (Part I & Part II) Placebo (Part I) Verubecestat (Part II) | Continued | 2, 3 |
| 29 | Thalidomide | Unknown |  |
| 30 | Dimebon | Completed |  |
| 31 | 3APS | Unknown |  |
| 32 | Aricept;  INM-176 | Completed |  |
| 33 | AC-3933; sugar pill | Completed |  |
| 37 | Octohydroaminoacridine Succinate Tablets | Unknown |  |
| 38 | LY450139 | completed |  |
| 40 | Bapineuzumab | Terminated |  |
| 41 | Interferon beta-1a; | Completed |  |
| 42 | ADENOSINE TRIPHOSPHATE | Active | 2 |
| 43 | ACC-001 (vanutide cridificar) | Completed |  |
| 45 | Rivastigmine | Completed |  |
| 46 | Resveratrol | Completed |  |
| 47 | Varenicline | Completed |  |
| 49 | Placebo and Nilotinib Capsule(s) | Continued | 2 |
| 50 | Benfotiamine | Continued | 2 |
| 51 | Acitretin | Unknown |  |
| 52 | Xaliproden (SR57746A) | Completed |  |
| 55 | Flutemetamol (18F) Injection | Completed |  |
| 56 | MABT5102A | Completed |  |
| 57 | TRx0237 150-250 mg/day | Completed |  |
| 58 | TRx0014 | Completed |  |
| 59 | Indomethacin | Completed |  |
| 60 | Memantine | Terminated |  |
| 61 | AH110690 (18F) Injection | Completed |  |
| 62 | Dietary Supplement: Curcumin Formulation; | Unknown |  |
| 63 | Solanezumab | Active | 3, 4 |
| 67 | AChEI,  RO4602522 1 mg; RO4602522 5 mg; | Completed |  |
| 69 | VI-1121 | Completed |  |
| 70 | Rrilapladib | Completed |  |
| 71 | Leuprolide acetate | Completed |  |
| 72 | Simvastatin | Completed |  |
| 73 | BAC |  | 2 |
| 74 | Nilvadipine | Active | 2 |
| 75 | PF-04447943 | Completed |  |
| 78 | RVT-101 |  | 3 |
| 79 | Azeliragon |  | 3 |


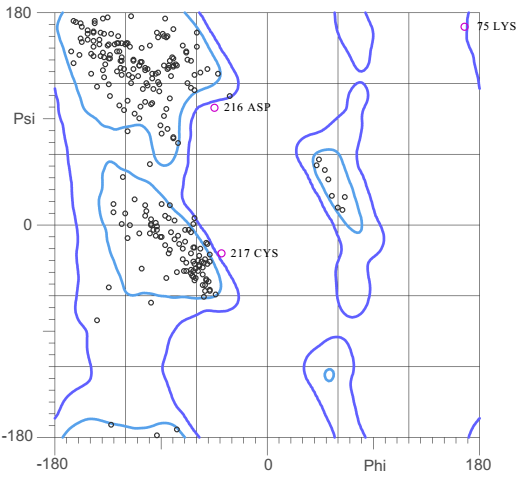


**Figure S2.** Ramachandran plot of BACE1 (PDBID: 2ZHV)

**
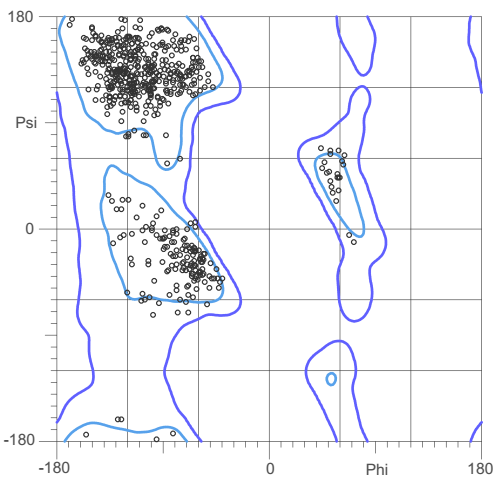
**

**Figure S3.** Ramachandran plot of solanezumab (PDBIDs: 4XXD)

**Table S4.** Structural analysis of BACE1, Aβ and Solanezumab

| **Metrics** | **BACE1** | **Aβ** | **Solanezumab** |
| --- | --- | --- | --- |
| Rfree | 0.290 | - | 0.312 |
| Clashscore | 14 | 27 | 8 |
| Ramachandran outliers | 1.1% | 3% | 0% |
| Side chain outliers | 1.0% | 30.9% | 1.9% |
| RSRZ outliers | 13.5% | - | 6.8% |

**
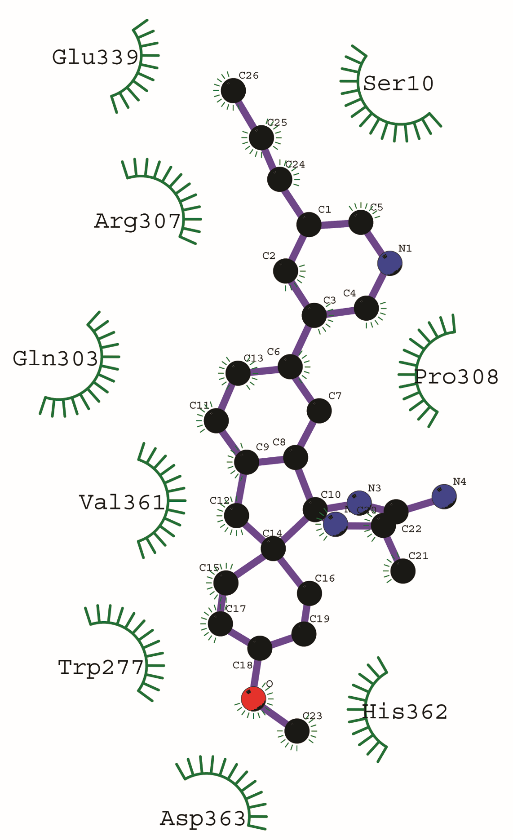
**

**Figure S5:** Docking pose 1 of AZD3293 against BACE1

**
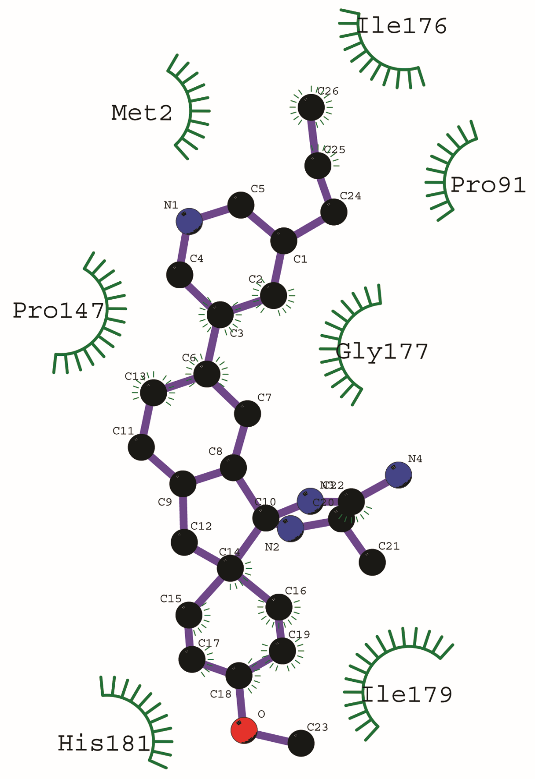
**

**Figure S6:** Docking pose 2 of AZD3293 against BACE1

**
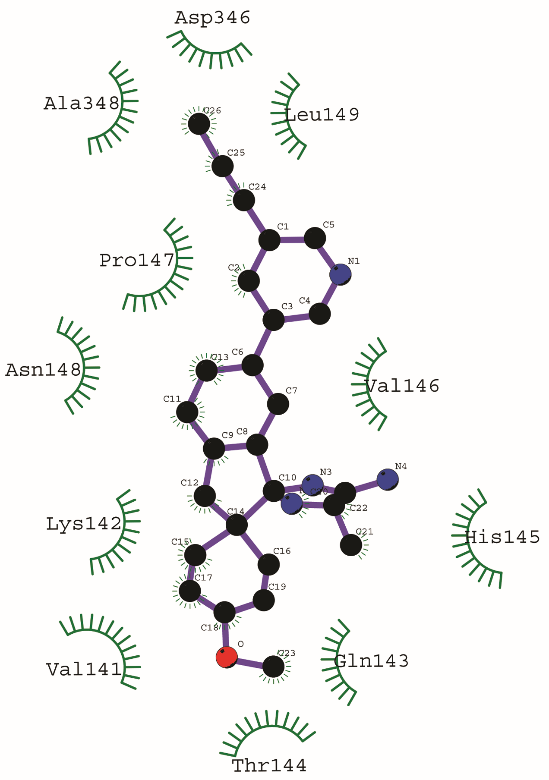
**

**Figure S7:** Docking pose 3 of AZD3293 against BACE1

**
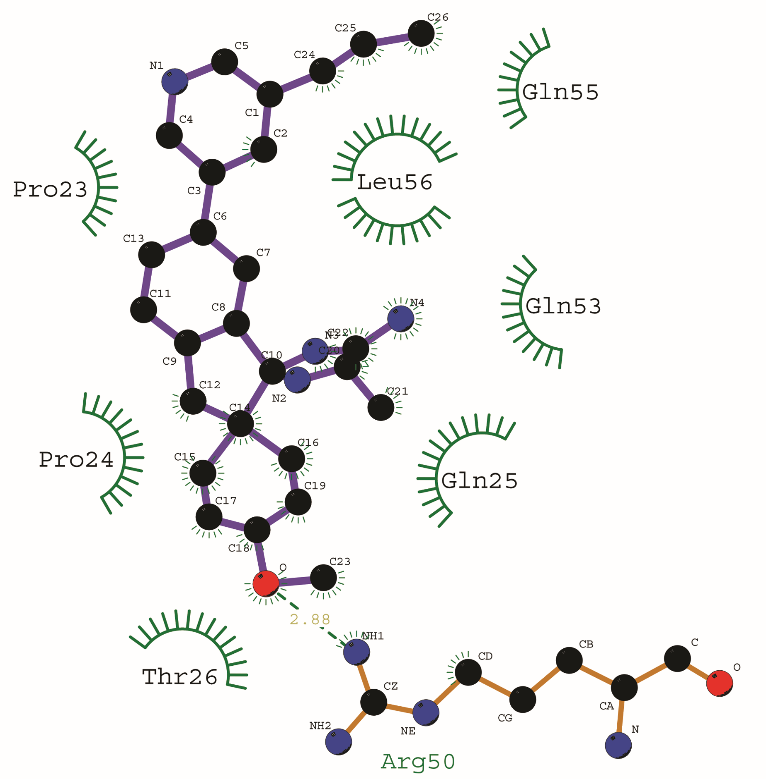
**

**Figure S8:** Docking pose 4 of AZD3293 against BACE1

**
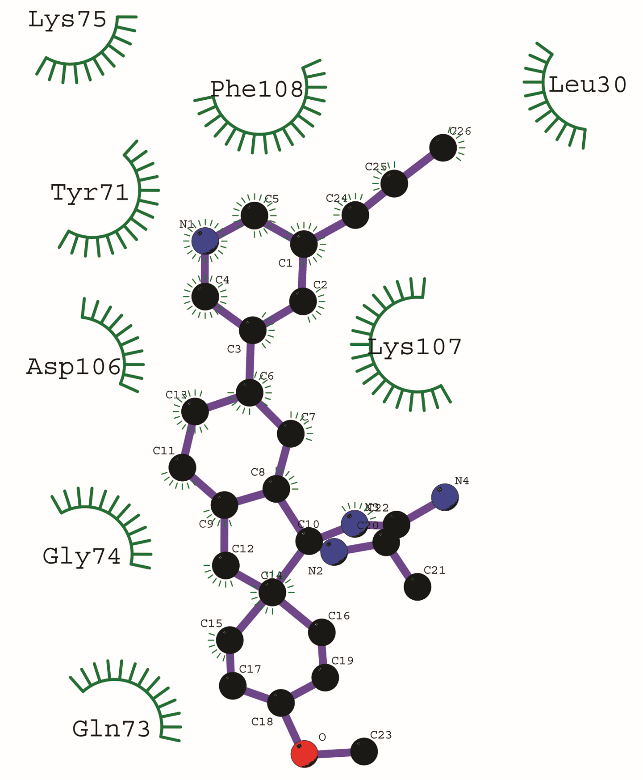
**

**Figure S9:** Docking pose 5 of AZD3293 against BACE1

**
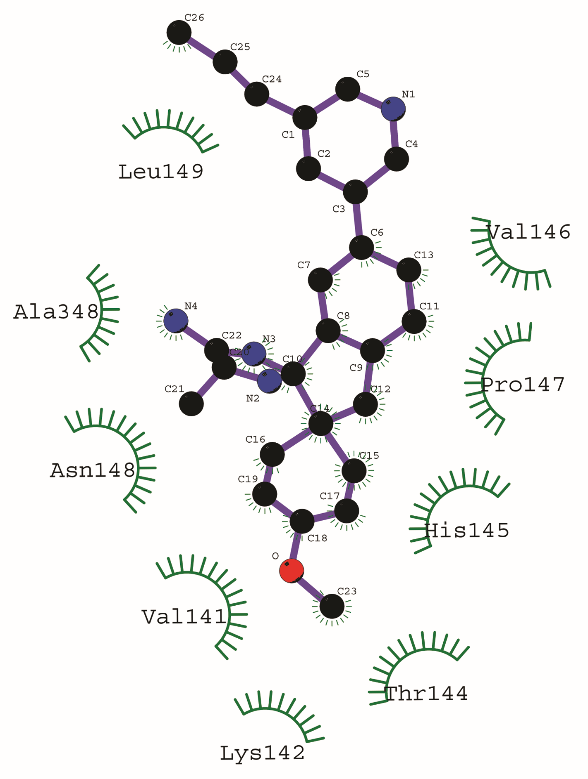
**

**Figure S10:** Docking pose 6 of AZD3293 against BACE1

**
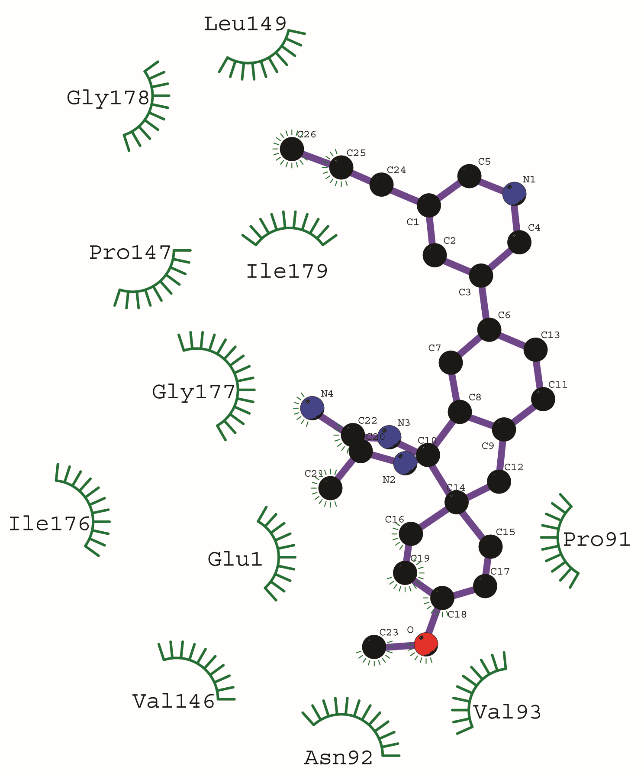
**

**Figure S11:** Docking pose 7 of AZD3293 against BACE1

**
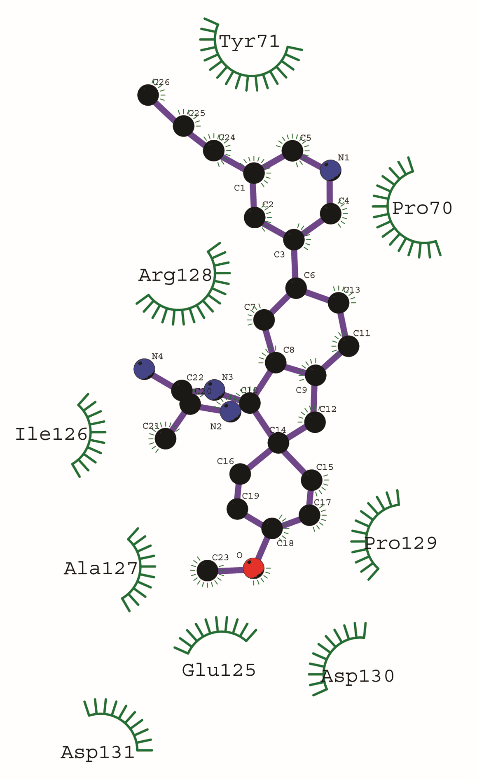
**

**Figure S12:** Docking pose 9 of AZD3293 against BACE1

**
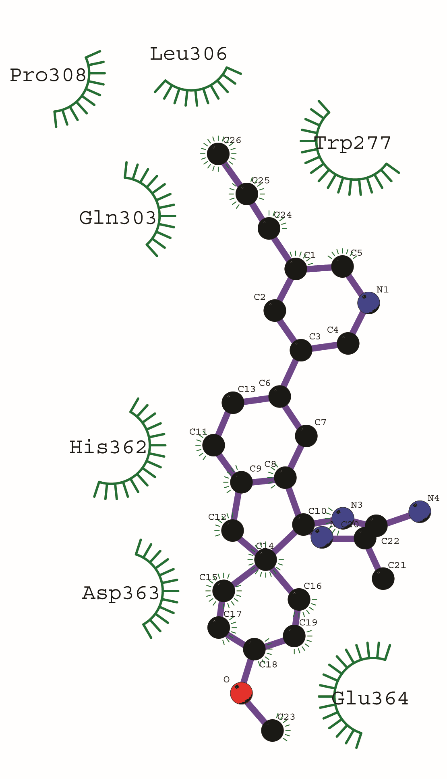
**

**Figure S13:** Docking pose 10 of AZD3293 against BACE1

**
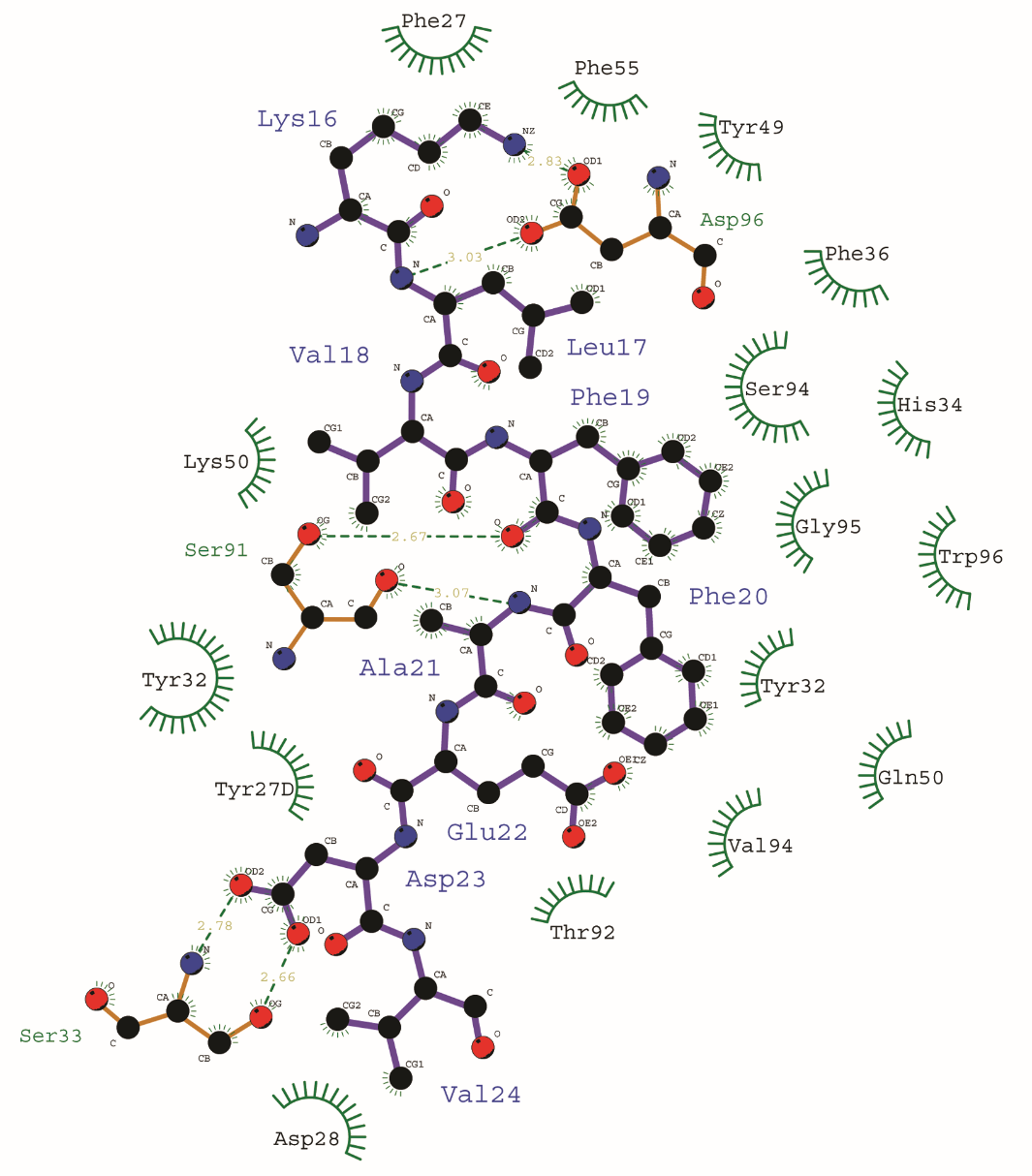
**

**Figure S14:** Binding interaction of Solanezumab with Aβ peptide
